# Sex-specific reference intervals of hematologic and biochemical analytes in *Sprague-Dawley* rats using the nonparametric rank percentile method

**DOI:** 10.1371/journal.pone.0189837

**Published:** 2017-12-20

**Authors:** Qili He, Guoming Su, Keliang Liu, Fangcheng Zhang, Yong Jiang, Jun Gao, Lida Liu, Zhongren Jiang, Minwu Jin, Huiping Xie

**Affiliations:** 1 Institute of Toxicological Detection, Sichuan Center for Disease Control and Prevention, Chengdu, Sichuan, China; 2 Department of Pharmacy and Laboratory, Sichuan Nursing Vocational College, Chengdu, Sichuan, China; 3 Department of Ultrastructural Pathology Center, Renmin Hospital of Wuhan University, Wuhan, Hubei, China; Oregon State University, UNITED STATES

## Abstract

**Background:**

Hematologic and biochemical analytes of *Sprague-Dawley* rats are commonly used to determine effects that were induced by treatment and to evaluate organ dysfunction in toxicological safety assessments, but reference intervals have not been well established for these analytes. Reference intervals as presently defined for these analytes in *Sprague-Dawley* rats have not used internationally recommended statistical method nor stratified by sex. Thus, we aimed to establish sex-specific reference intervals for hematologic and biochemical parameters in *Sprague-Dawley* rats according to Clinical and Laboratory Standards Institute C28-A3 and American Society for Veterinary Clinical Pathology guideline.

**Methods:**

Hematology and biochemistry blood samples were collected from 500 healthy *Sprague-Dawley* rats (250 males and 250 females) in the control groups. We measured 24 hematologic analytes with the Sysmex XT-2100i analyzer, 9 biochemical analytes with the Olympus AU400 analyzer. We then determined statistically relevant sex partitions and calculated reference intervals, including corresponding 90% confidence intervals, using nonparametric rank percentile method.

**Results:**

We observed that most hematologic and biochemical analytes of *Sprague-Dawley* rats were significantly influenced by sex. Males had higher hemoglobin, hematocrit, red blood cell count, red cell distribution width, mean corpuscular volume, mean corpuscular hemoglobin, white blood cell count, neutrophils, lymphocytes, monocytes, percentage of neutrophils, percentage of monocytes, alanine aminotransferase, aspartate aminotransferase, and triglycerides compared to females. Females had higher mean corpuscular hemoglobin concentration, plateletcrit, platelet count, eosinophils, percentage of lymphocytes, percentage of eosinophils, creatinine, glucose, total cholesterol and urea compared to males. Sex partition was required for most hematologic and biochemical analytes in *Sprague-Dawley* rats. We established sex-specific reference intervals, including corresponding 90% confidence intervals, for *Sprague-Dawley* rats.

**Conclusions:**

Understanding the significant discrepancies in hematologic and biochemical analytes between male and female *Sprague-Dawley* rats provides important insight into physiological effects in test rats. Establishment of locally sex-specific reference intervals allows a more precise evaluation of animal quality and experimental results of *Sprague-Dawley* rats in our toxicology safety assessment.

## Introduction

Reference intervals are very important laboratory tools used to make a medical diagnosis, therapeutic management decision, or other physiological assessment in the clinical laboratory [1]. This is an important task for clinical laboratories to provide reliable reference intervals, which are defined as the 2.5^th^ and 97.5^th^ percentiles of healthy individual distributions [1, 2]. To ensure appropriate interpretation of test results, it is necessary that the reference intervals were stratified by key covariates such as age, sex, pregnancy, geographic location and ethnic background [2–8]. Clinical and Laboratory Standards Institute (CLSI) document C28-A3 is a standard for defining, establishing and verifying reference intervals in the clinical laboratory [1]. In addition, the American Society for Veterinary Clinical Pathology (ASVCP) has issued a consensus guideline for determination of de novo reference intervals in veterinary species [2], which mirror the 2008 CLSI recommendations.

Over the last decade, as a result of toxicology and toxicological pathology rapid expansion, pre-clinical toxicity and safety studies have become more important. Therefore, a license must be issued before marketing any new drugs, additives, food, or new chemical products [9]. *Sprague-Dawley* rats have been widely used in toxicological safety studies [10–12]. Hematology and biochemistry are commonly used to determine the biological significance of the findings that cannot be detected by direct examination of organs and tissues in toxicity and safety studies. Hematologic and biochemical reference values are critical for assessing the health and disease states associated with the diagnosis of blood disorders, infectious diseases, immune system and lipoprotein metabolism, glucose regulation, and liver and kidney function. When these parameters have deviated from their normal homeostatic state, the increase or decrease of their activities or concentrations could result in pathology [13].

Several recent studies determined hematology and biochemistry reference intervals in *Wistar* rats [14], *C57BL/6J* mice [15, 16], Pine voles [17], Monkeys [18], Long-tailed macaques [19], Straw-colored fruit bats [20] and Atlantic sturgeon [21]. Despite different reference values for *Sprague-Dawley* rats have been provided in some studies [5, 6, 9, 22]. The values are more than 5 years and can be affected by many factors, such as nutrition, animal housing, or beverage. In addition, these publications lack sufficient data for a detailed analysis for *Sprague-Dawley* rats stratifying by sex, and use the mean and standard deviations for establishing reference intervals, which are not the CLSI and ASVCP recommendations.

Therefore, it is imperative to take into account the various influences on the measured laboratory test results, by using a more systematic process to develop appropriate local reference intervals for *Sprague-Dawley* rats. According to CLSI C28-A3 guideline and ASVCP guideline, using a strict quality control program, we intended to establish reference intervals for hematologic and biochemical parameters in *Sprague-Dawley* rats by using the nonparametric rank percentile method.

## Materials and methods

### Ethics statement

This study was approved by the Ethics Committee of Sichuan Center for Disease Control and Prevention (Approval ID 2016–27). All experiments were conducted under pentobarbital sodium anesthesia in order to minimize any suffering of experimental animals.

### Animal care and management

Four-week-old *Sprague-Dawley* rats were provided by the Traditional Chinese Medicine Academy of Sciences Laboratory Animal Center (Chengdu, China). The animals were acclimated and quarantined for 1 week prior to the initiation of a repeated dose 28-day oral toxicity study and their health status had been evaluated. Animals were randomly divided into control group and treatment groups. Each consisted of ten male and female rats. The animals were given test substance by gavage for a period of 28 consecutive days. The body weight of all rats was recorded at baseline and then was noted weekly until their scheduled necropsy. Body weight results were expressed as mean ± standard deviation. At the beginning of treatment, the rats were around 5 weeks of age. Body weight was 74.71 ± 10.42g for males and 76.26 ± 10.22g for females.

All rats were individually housed in cages. Barrier system animal room controls were set to maintain temperature at 22 ± 2°C and relative humidity 40% to 70%, with a 12-hour day-night cycle with artificial light and ≥ 12 times/hour of ventilation frequency. Drinking water and full nutritional granular feed were supplied ad libitum to the animals.

The repeated dose 28-day oral toxicity study was conducted in accordance with National Food Safety Standards of People’s Republic of China GB 15193.22 [23] and the Organization for Economic Cooperation and Development Testing Guideline No.407 [24].

### Samples

Hematology and biochemistry samples for this study obtained from control group *Sprague-Dawley* rats. Control group rats were fed with an equivalent volume of distilled water. A total of 500 rats (250 males and 250 females) were collected as reference individuals from May to December 2016. At the end of the stipulated experimental period, animals were fasted for approximately 16 hours and received water ad libitum. Animals were anesthetized and euthanized with an intraperitoneal injection of pentobarbital sodium (60mg/kg). The animals were kept at room temperature for 15 to 25min after the administration to induce anesthesia. Blood was taken from the abdominal vena cava and collected as follows: 1ml in a purple Ethylene Diamine Tetraacetic Acid-2K tube for complete blood count and mixed to avoid clotting, 3ml in a plain tube for chemistry testing, then left for about 30 minutes at room temperature and centrifuged at 3500g for 15 min to harvest serum. Hematology and biochemistry samples were processed within 2 hours of the blood being drawn. Samples that showed clotting, platelets clumping, or hemolysis were not used because the analyte being measured could be affected.

The selected tissues and organs from the control group animals were collected and preserved in 10% neutral buffered formalin. All processed tissues from the control group were embedded in paraffin, sectioned approximately 3μm thick, stained with hematoxylin and eosin, and examined microscopically for histopathology.

### Calibration and assurance of quality control

Routine maintenance, calibration, quality control (QC), verification of the Sysmex XT-2100i analyzer and Olympus AU400 analyzer were performed to ensure accuracy and precision. Calibration and QC materials included Sysmex SCS-100 and 2 levels of e-check controls (Level 1 and Level 2), Beckman Coulter system calibrator and control serum 1 and 2. Each analyte internal quality control (IQC) performance was tracked using real-time Levey Jennings QC plots monitored within 2 standard deviations of the laboratory established control mean. The external quality assurance (EQA) involved proficiency testing using the National Institutes for Food and Drug Control (NIFDC) PT-090 surveys performed for biochemistry once a year, as well as National Institute of Measurement and Testing Technology (NIMTT) calibration surveys for hematology. Within-day precision was calculated based on testing samples from healthy individual 20 times within the same day while between-day precision was assessed by measuring controls over consecutive days from May to December 2016.

### Hematology and biochemistry analyte tests

Hematology and biochemistry samples were analyzed with automated blood cell counter Sysmex XT-2000i and Olympus AU 400 analyzers right after drawing blood. The 24 hematology parameters included hemoglobin, hematocrit, mean corpuscular hemoglobin (MCH), mean corpuscular hemoglobin concentration (MCHC), mean corpuscular volume (MCV), red blood cell (RBC) count, red cell distribution width (RDW), plateletcrit, platelet distribution width (PDW), platelet count, platelet large cell ratio (P-LCR), mean platelet volume (MPV), white blood cell (WBC) count and a 5-part differential count (neutrophils, lymphocytes, monocytes, eosinophils and basophils), and percentages of 5-part differential counts.

Nine biochemistry parameters included alanine aminotransferase (ALT), albumin, aspartate aminotransferase (AST), creatinine, glucose, total cholesterol, total protein, triglycerides, and urea. Methods for all analysis were traceable to International Federation of Clinical Chemistry (IFCC) standards.

### Statistical analyses and determination of reference intervals

Participants were excluded from the statistical analysis on the basis of organs histopathology changes before calculation of reference intervals. Individual histograms for each hematology and biochemistry analyte in each subgroup were visually checked for outliers, and extreme values were handled according to the ratio D/R proposed by Dixon [25] and Reed et al [26]. After removing significant outliers, we assessed the normality of male and female subgroups with Kolmogorov-Smirnov test. Box and whisker plots were applied to check differences between genders. We then calculated reference intervals by using nonparametric rank percentile method determinations of the 2.5^th^ and 97.5^th^ individual percentiles, as well as 90% confidence intervals for the upper and lower limits of each reference interval, including male group, female group, and combined group. If the data were not skewed, then we stratified the subjects according to sex using the Harris-Boyd test [27]. Conversely, if data were found to be skewed, the Lahti algorithm method [28] was used to assess the need for partitioning the reference intervals. We also compared the generated reference intervals with other results from different species. All calculations were performed in accordance with the CLSI C28-A3 and ASVCP guideline. Statistical analysis was performed with SPSS 16.0 and R Studio software, and a two tailed *P*-value < 0.05 was considered to be statistically significant.

## Results

### Participant samples

**Fig 1:**
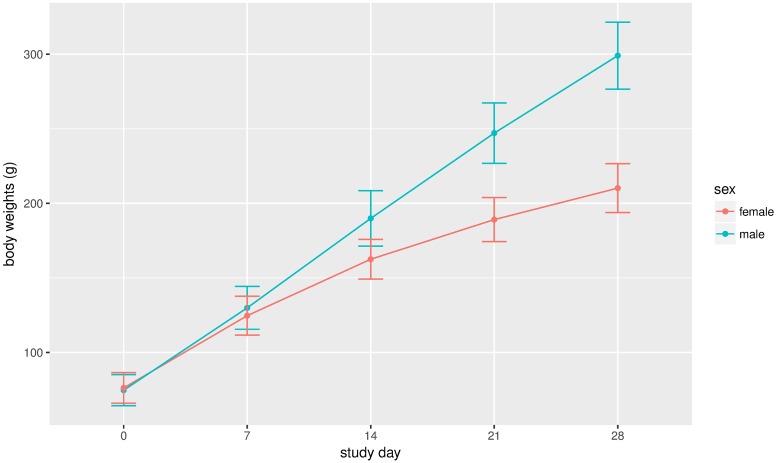
Mean weekly body weight (mean ± standard deviation) of control group *Sprague-Dawley* rats during the repeated dose 28-day oral toxicity study. Male rats were indicated by a green line, and female rats were indicated by a red line.

**Fig 2:**
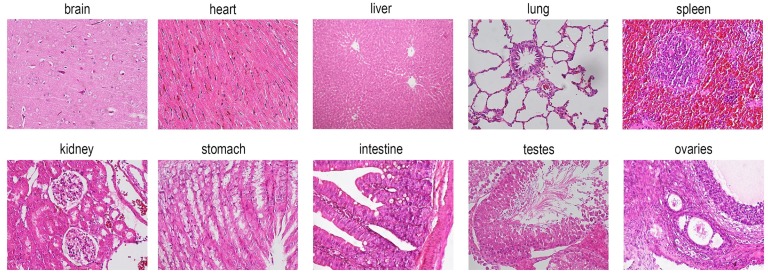
The histopathology of organs from the control group *Sprague- Dawley* rats (200×magnification). No significant damage was observed in the brain, heart, liver, lung, spleen, kidney, stomach, intestine, testes or ovaries of rats.

At the time of sacrifice, male and female rats were about 9 weeks old and weighed 299.06 ± 22.46g and 210.21 ± 16.40g respectively (Fig 1). As showed in Fig 2, no apparent histopathological abnormalities or lesions were observed from the control *Sprague-Dawley* rats. All data came from healthy participants, and no data were eliminated as statistical outliers in our study. 500 participant samples (250 males and 250 females) were used to calculate reference intervals.

### Quality control

The IQC performance was acceptable for all parameters processed over the study period. EQC tested met the performance evaluation criteria of both NIFDC and NIMTT programs. Precision of the repeats and differences between the split controls did not exceed the limits shown in S1 Table, indicating that the quality of performance was satisfactory for the Sysmex XT-2000i and Olympus AU 400 analyzers. Measurement method details were provided in S1 Table as well.

### Reference intervals of hematologic and biochemical analytes

**Fig 3:**
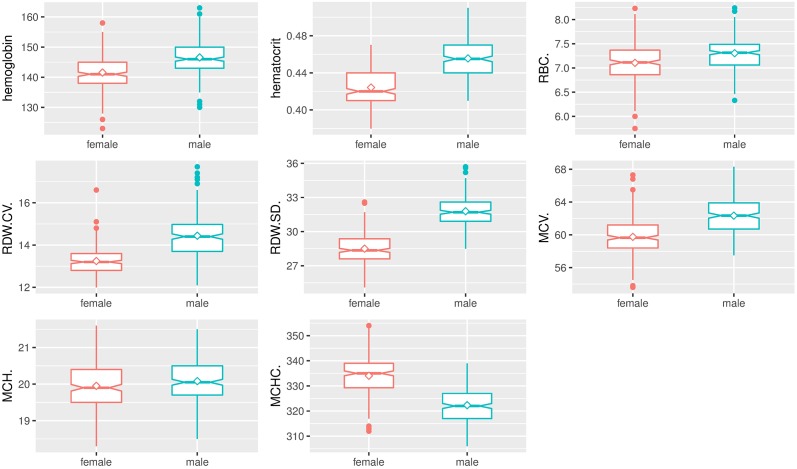
Box and whisker plots show differences in analyte levels of male and female *Sprague-Dawley* rats on RBC parameters. Data were depicted as box plots that indicated the median, 25^th^ and 75^th^ percentiles (box), mean (◇), and 1.5 times of interquartile (whiskers). Male rats were indicated by a green line, and female rats were indicated by a red line.

**Fig 4:**
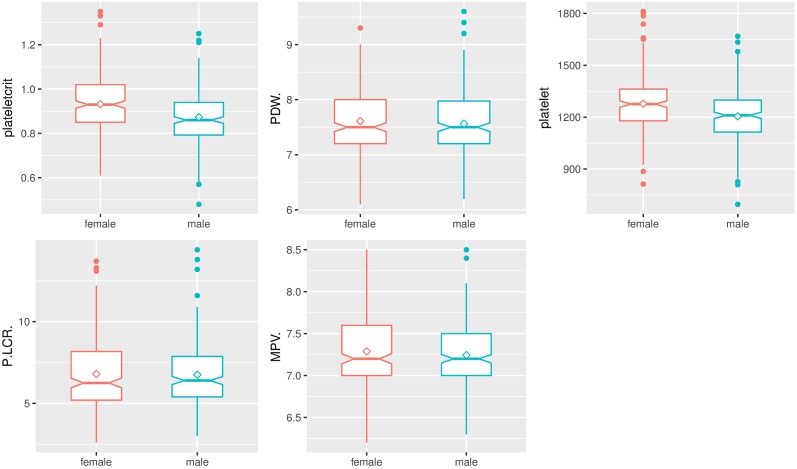
Box and whisker plots show differences in analyte levels of male and female *Sprague-Dawley* rats on platelet parameters. Data were depicted as box plots that indicated the median, 25^th^ and 75^th^ percentiles (box), mean (◇), and 1.5 times of interquartile (whiskers). Male rats were indicated by a green line, and female rats were indicated by a red line.

**Fig 5:**
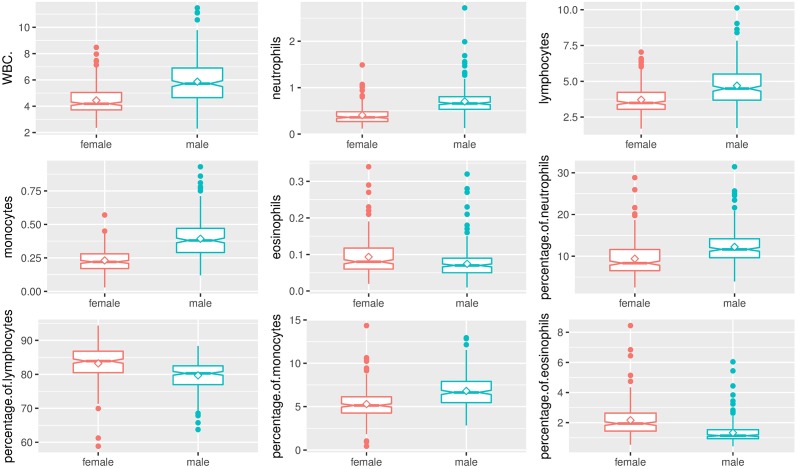
Box and whisker plots show differences in analyte levels of male and female *Sprague-Dawley* rats on WBC parameters. Data were depicted as box plots that indicated the median, 25^th^ and 75^th^ percentiles (box), mean (◇), and 1.5 times of interquartile (whiskers). Male rats were indicated by a green line, and female rats were indicated by a red line.

**Fig 6:**
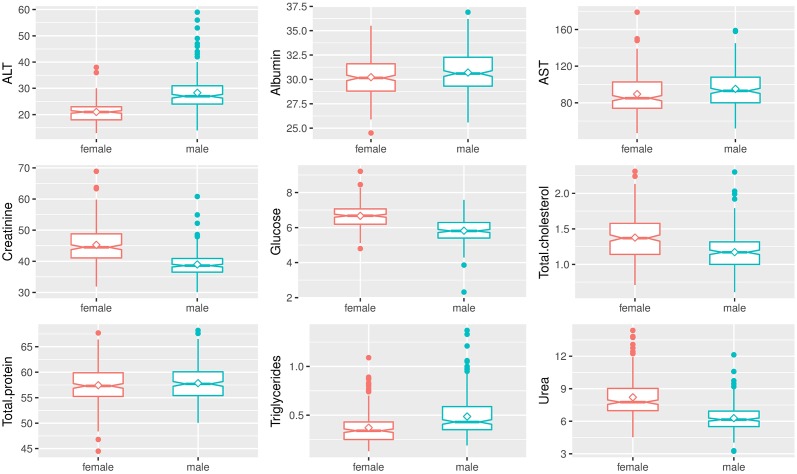
Box and whisker plots show differences in analyte levels of male and female *Sprague-Dawley* rats on biochemical parameters. Data were depicted as box plots that indicated the median, 25^th^ and 75^th^ percentiles (box), mean (◇), and 1.5 times of interquartile (whiskers). Male rats were indicated by a green line, and female rats were indicated by a red line.

As expected, box and whisker plots indicated the marked effect of sex on hematologic and biochemical parameters of *Sprague-Dawley* rats (Figs 3–6). Male rats had significantly higher hemoglobin, hematocrit, RBC count, RDW, MCV, MCH, WBC count, neutrophils, lymphocytes, monocytes, percentage of neutrophils, percentage of monocytes, ALT, AST, and triglycerides compared to female rats. Female rats had significantly higher MCHC, plateletcrit, platelet count, eosinophils, percentage of lymphocytes, percentage of eosinophils, creatinine, glucose, total cholesterol, and urea compared to male rats. No significant differences between male and female rats were found in MPV, PDW, P-LCR, albumin, and total protein.

According to results of standard normal deviate test (z value > critical z value of 5) and proportions of the subgroups outside each of the reference limits of the combined distribution (proportion criteria), as well as appropriate clinical consideration, partitioning of reference intervals were recommended for hemoglobin, hematocrit, RDW-CV, RDW-SD, MCV, MCHC, WBC count, neutrophils, lymphocytes, monocytes, percentage of neutrophils, percentage of lymphocytes, percentage of monocytes, percentage of eosinophils, ALT, creatinine, glucose, total cholesterol, triglycerides and urea based on sex (S2 and S3 Tables). Sex-specific reference intervals for hematologic and biochemical analytes, including corresponding 90% confidence intervals, were provided in Tables 1 and 2.

**Table 1:** Sex-specific reference intervals for 24 hematologic analytes in *Sprague-Dawley* rats.

Analyte	Sex	Median	Reference interval	Lower Ref Lim 90% CI	Upper Ref Lim 90% CI
Hemoglobin, g/L	Male	146	135–159	131–139	157–161
	Female	141	129–154	126–132	151–155
Hematocrit, %	Male	46	42–49	41–43	48–50
	Female	42	40–46	39–40	45–47
RBC, 10^12^/L	Combined	7.21	6.39–8.01	6.35–6.45	7.88–8.05
RDW-CV, %	Male	14.40	13.03–16.57	12.70–13.20	16.20–17.40
	Female	13.20	12.23–14.57	12.00–12.30	14.40–15.10
RDW-SD, fL	Male	31.7	29.50–34.55	28.50–29.80	34.10–35.60
	Female	28.35	25.90–31.37	25.50–26.20	31.00–32.50
MCV, fL	Male	62.35	58.01–67.00	57.60–58.80	66.50–67.90
	Female	59.65	55.21–64.80	53.60–56.00	63.90–66.80
MCH, pg	Combined	20.00	18.70–21.20	18.70–18.80	21.20–21.30
MCHC, g/L	Male	322	310–336	308–310	334–337
	Female	335	318–347	313–320	345–353
Plateletcrit, %	Combined	0.90	0.65–1.16	0.63–0.67	1.14–1.22
PDW, fL	Combined	7.50	6.75–8.85	6.70–6.80	8.80–8.90
Platelet, 10^9^/L	Combined	1240	923–1580	879–961	1533–1632
P-LCR, %	Combined	6.30	4.05–11.20	3.60–4.20	10.80–12.20
MPV, fL	Combined	7.20	6.70–8.10	6.60–6.70	8.00–8.30
WBC, 10^9^/L	Male	5.72	3.00–9.22	2.35–3.57	8.60–11.13
	Female	4.19	2.58–7.34	2.40–2.76	6.80–7.96
Neutrophils, 10^9^/L	Male	0.66	0.28–1.43	0.24–0.33	1.17–1.99
	Female	0.36	0.19–0.91	0.15–0.20	0.79–1.07
Lymphocytes, 10^9^/L	Male	4.49	2.45–7.66	1.76–2.76	7.06–9.04
	Female	3.49	2.09–6.39	1.83–2.25	5.77–6.59
Monocytes, 10^9^/L	Male	0.38	0.17–0.76	0.14–0.20	0.67–0.86
	Female	0.22	0.08–0.43	0.04–0.10	0.39–0.45
Eosinophils, 10^9^/L	Combined	0.07	0.03–0.21	—	0.18–0.25
Basophils, 10^9^/L	Combined	0.00	0–0.02	—	—
Neutrophils, %	Male	11.64	6.14–22.95	4.54–6.54	20.44–25.64
	Female	8.34	4.27–18.48	3.84–4.54	17.34–25.94
Lymphocytes, %	Male	80.29	69.68–86.89	65.74–71.54	86.24–88.14
	Female	83.89	71.77–89.94	61.24–74.84	89.34–91.74
Monocytes, %	Male	6.64	3.77–10.82	2.84–4.14	10.54–12.84
	Female	5.14	2.10–9.34	0.94–2.84	8.24–10.64
Eosinophils, %	Male	1.14	0.54–3.39	0.44–0.64	2.64–5.44
	Female	1.94	0.84–4.29	0.64–0.94	3.94–6.84
Basophils, %	Combined	0.04	0.04–0.44	—	0.34–0.64

Combined indicate a combination of male and female. Ref Lim, Reference Limit; CI, confidence intervals.

**Table 2:** Sex-specific reference intervals for 9 biochemical analytes in *Sprague-Dawley* rats.

Analyte	Sex	Median	Reference interval	Lower Ref Lim 90% CI	Upper Ref Lim 90% CI
ALT, U/L	Male	27	19–47	18–20	43–56
	Female	21	14–30	13–16	28–36
Albumin, g/L	Combined	30.40	26.85–34.55	26.70–27.20	34.20–34.90
AST, U/L	Combined	89	60–139	58–62	134–144
Creatinine, μmol/L	Male	38.60	32.36–47.90	31.30–33.40	46.00–54.90
	Female	44.50	34.91–59.67	33.00–37.00	57.60–63.70
Glucose, mmol/L	Male	5.82	4.46–7.24	3.86–4.76	6.93–7.49
	Female	6.67	5.31–8.01	5.12–5.61	7.80–8.45
Total protein, g/L	Combined	57.50	51.10–64.55	50.60–51.50	64.20–65.50
Total cholesterol, mmol/L	Male	1.17	0.68–1.77	0.63–0.75	1.67–2.03
	Female	1.38	0.81–2.03	0.74–0.85	1.93–2.24
Triglycerides, mmol/L	Male	0.43	0.23–0.99	0.19–0.25	0.90–1.33
	Female	0.34	0.16–0.89	0.13–0.18	0.80–0.89
Urea, mmol/L	Male	6.16	4.32–8.97	3.28–4.54	8.63–10.59
	Female	7.76	5.56–12.67	5.31–5.86	11.71–13.82

Combined indicate a combination of male and female. Ref Lim, reference limit; CI, confidence intervals.

### Comparison of reference intervals

A comparison of the generated reference intervals with results from *Wistar* rats [8, 14, 29], *C57BL/6J* mice [14, 16] and humans [30–34] was presented in Table 3, detailed sex-specific reference intervals showed in most analytes.

**Table 3:** Reference intervals for key blood variables of different species.

Analyte	Sex	Species			
		*Sprague-Dawley* rats	*Wistar* rats^a^	*C57BL/6J* mice^b^	Humans^c^
Hemoglobin, g/L	Male	135–159	91–103	41–168	130–175
	Female	129–154	88–101	57–170	115–150
Hematocrit, %	Male	42–49	42–48	12.5–53.0	40–50
	Female	40–46		16.2–58.3	35–45
RBC, 10^12^/L	Male	6.39–8.01	8.20–9.50	2.93–11.01	4.30–5.80
	Female		7.80–9.00	3.47–11.73	3.80–5.10
MCV, fL	Male	58.01–67.00	48–54	—	82–100
	Female	55.21–64.80	48–57	—	
MCH, pg	Male	18.70–21.20	16.10–19.30	—	27–34
	Female		17.70–19.30	—	
MCHC, g/L	Male	310–336	340–361	—	316–354
	Female	318–347	324–359	—	
Platelet, 10^9^/L	Male	923–1580	573–998	156–1037	125–350
	Female		591–836	144–894	
MPV, fL	Male	6.70–8.10	6.70–8.00	—	7–11
	Female		7.00–7.80	—	
WBC, 10^9^/L	Male	3.00–9.22	3.40–9.50	2.47–14.42	3.50–9.50
	Female	2.58–7.34	2.20–5.90	2.20–11.53	
Neutrophils, 10^9^/L	Male	0.28–1.43	0.40–1.50	—	1.80–6.30
	Female	0.19–0.91	0.30–1.00	—	
Lymphocytes, 10^9^/L	Male	2.45–7.66	2.60–7.80	—	1.10–3.20
	Female	2.09–6.39	1.70–4.80	—	
Monocytes, 10^9^/L	Male	0.17–0.76	—	—	0.10–0.60
	Female	0.08–0.43	0.10–0.40	—	
Eosinophils, 10^9^/L	Male	0.03–0.21	0–0.20	—	0.02–0.52
	Female		0–0.10	—	
Basophils, 10^9^/L	Combined	0–0.02	0–0.04	—	0–0.06
ALT, U/L	Male	19–47	24–49	46–70	9–50
	Female	14–30	23–67	42–73	7–40
Albumin, g/L	Male	26.85–34.55	44.40–58.40	22–42	40–55
	Female			20–47	
AST, U/L	Male	59–139	50–96	55–91	15–40
	Female		61–153	51–122	15–35
Creatinine, μmol/L	Male	32.36–47.90	31–48	8.80–13.20	57–97
	Female	34.91–59.67	37–53		41–73
Glucose, mmol/L	Male	4.46–7.24	5.10–9.20	5.60–9.10	3.89–6.11
	Female	5.31–8.01	5.70–8.40	5.20–12.20	
Total protein, g/L	Male	51.10–64.55	40–60	47–72	65–85
	Female		40–80	45–83	
Total Cholesterol, mmol/L	Male	0.68–1.77	1.10–2.00	1.80–3.90	2.80–5.20
	Female	0.81–2.03	0.70–2.50	1.30–3.40	
Triglycerides, mmol/L	Male	0.23–0.99	0.40–2.10	0.75–2.57	0.56–1.70
	Female	0.16–0.89	0.40–3.40	0.50–1.76	
Urea, mmol/L	Male	4.32–8.97	4.00–9.30	3.20–13.20	3.10–8.00
	Female	5.56–12.67	6.80–11.30	6.00–11.50	2.60–7.50

Combined indicate a combination of male and female.

^a^Reference data for *Wistar* rats are from references [8, 14, 29].

^b^Reference data for *C57BL / 6J* mice are from references [14, 16].

^c^Reference data for humans are from references [30–34].

Rodents (*Sprague-Dawley* rats, *Wistar* rats and *C57BL/6J* mice) showed markedly higher reference intervals for RBC and platelet than in humans. WBC counts were similar between rodents and humans, but rodents had higher lymphocyte reference intervals and lower neutrophil reference intervals compared to humans. Rodents also displayed higher AST, glucose and urea reference intervals compared to humans. Similarly, the lower limit of reference interval for ALT in *Sprague-Dawley* rat was significantly higher than that of humans (14 vs. 7 U/L in females, 19 vs. 9 U/L in males). Level of triglycerides in rodents was different.

In contrast to rodents, humans had higher reference intervals for MCV, MCH, albumin, creatinine, total cholesterol, and total protein. In particular, the reference interval of urea and creatinine in human males was significantly higher than that in females. But the opposite occurred in *Sprague-Dawley* and *Wistar* rats.

## Discussion

In this study, we establish sex-specific reference intervals of hematologic and biochemical analytes in control group *Sprague-Dawley* rats by using internationally accepted CLSI and ASVCP guidelines. It is important to note that most parameters are different between male and female groups.

Sex differences were observed in RBC parameters, the same trends were reported in previous studies [9, 35]. It might be due to the effect of testosterone which activates erythropoiesis by stimulating erythropoietin production [36]. For WBC, neutrophils, lymphocytes, and monocytes, the reference interval was significantly higher in males than that in females. The findings were in agreement with studies in *Sprague-Dawley* rats, *C57BL/J* mice, *C3H/HeJ* mice and *Wistar* rats [8, 9, 16]. However, higher lymphocytes and monocytes were reported in females than in males, when the rats were older than three months of age [8]. Leukocyte variations might be related to the development of immunocompetence and immune surveillance system and were affected by age [37, 38]. In addition, Petterino C et al. [9] showed that the WBC count was almost double compared to our results. This can be attributed to difference in anesthesia and venipuncture sites. Our data showed that platelets of females were significantly higher than those of males, a finding previously reported in humans [39, 40]. The sex-change for PCT exactly resembled platelets.

Concerning biochemical parameters, males generally showed higher values than females, in agreement with previously reported for ALT, AST and triglycerides [5, 22, 41], but not for creatinine, glucose, total cholesterol, and urea [5, 14, 41].

ALT and AST are classic and most widely hepatocellular injury markers, but not only specific for the liver. ALT has been also used as a biomarker of non-alcoholic fatty acid liver disease [42], and associated with insulin resistance, metabolic syndrome and type 2 diabetes [43]. Transaminase levels were higher in males than in females, because males had lower subcutaneous fat storage capacity, driven predominantly by sex hormone, thus excess fat quickly entered into intra-abdominal and perivascular cells, skeletal muscle, liver and pancreas, ALT and AST increased during this process [44].

Concentrations of the renal markers creatinine and urea are related to the glomerular filtration rate and protein degradation, respectively. The fraction of excreted creatinine can accurately determine the glomerular filtration rate. Renal organic anion transporter 1 mRNA expression was higher in male than in female mice, reflecting significantly the higher fraction of creatinine secretion in males, which most likely contributed to the low plasma creatinine levels generally found in male mice [45]. Another study has reported that creatinine concentration increase in female rats after 16 hours of fasting [46]. Higher urea concentration in females may be due to different substrate energy utilization under different physiological conditions, as well as the effect of gender on amino acid catabolism [47].

The lipid markers, total cholesterol and triglycerides, reflect cardiovascular risk factors. Low HMG-CoAR expression in female indicated that the neo-synthesis of cholesterol was physiologically lower than male [48]. However, in this study, total cholesterol of female rats was higher than that of male rats. This might be explained by physiological events developed at an early stage of life has the sex-related cholesterol metabolism [49], such as absorption, synthesis and catabolism. Another study showed that testosterone suppresses plasma cholesterol levels in animals when fed a hypercholesterolemic diet [50]. For glucose, females were at higher concentrations than males, similar to the results of fasting for 16 hours [46]. This likely reflects differences in energy demands, basal insulin secretion and insulin sensitivity between male and female. Insulin/blood glucose ratios were higher in males compared with females [51]. Increased glucose clearance might lead to increasing liver and muscle glycogen synthesis, conversion of glucose into triglycerides and increase fat deposition [52].

Compared with other studies, some reference values displayed major discrepancies between rodents and humans. We had no direct statistical comparison for other *Sprague-Dawley* rats, because the earlier studies reported only means±standard deviation, whereas we defined the reference interval using the 2.5^th^ and 97.5^th^ percentiles.

Owing to the shorter life span of RBC in mice and rats, production of RBC in times of increased demand preferentially occurred in the spleen, as the spleen in murine was more efficient than bone marrow in RBC production [53, 54]. This may be the reason why rodents show a higher reference interval for RBC count than humans. Neutrophils and lymphocytes were similar in rodents, which had predominantly more lymphocytes than neutrophils, but the opposite occurred in humans. This might be the maturation of the immune system in rats is delayed relative to humans [55], the immature innate immune system of rodents effort to produce rapid responding cells during a time period when the adaptive immune system is not yet highly contributory [56].

Murine platelets were similar to humans in platelets functionally, but they were at least approximately four to five times more numerous, smaller in size, much shorter platelet lifespan [57, 58]. Although humans and murine megakaryocytes displayed similar ploidy, the greater number of platelets in murine might be possibly due to the higher frequency of megakaryocytes per surface unit in histological sections of mouse bone marrow than in humans [57]. It is well known that bone marrow was a major site of platelet production, but murine megakaryocytes were also consistently localized in the spleen [59, 60]. The latest research has found that a large number of megakaryocytes circulate through the lungs in mice. And lung is the primary site of terminal platelet production, which accounting for approximately 50% of total platelet [61].

Because of the differences in anatomy such as lack of gallbladder in rats, and diverse metabolic liver function might also explain the differences in transaminase levels between rodents and humans [14]. Daily repetition of handling procedures and stress may also cause transient increase in ALT and AST. This may be another reason why ALT and AST show higher activity in rodents. During toxicity studies, increased ALT and AST, generally AST > ALT, could be considered as a result of intramuscular anesthetize and other handling procedures [13]. Haemolysis could raise the level of AST consequently, but our samples were almost free of hemolysis.

Rodents showed higher values of plasma glucose and urea, lower albumin, creatinine, total cholesterol, and total protein compared to humans. Higher glucose in rodents has been reported in another study [62]. Glucose and plasma lipid levels were partially genetically determined, which involved multiple genes [63, 64]. Higher level of urea in rodents could be conserved water more effectively by increasing renal concentrating ability [65]. The variation of albumin and total protein between humans and rodents may vary based on nutritional status or other physiological condition. Creatinine levels in rodents were low, which agreed with the results of other previous studies [14]. In humans, high creatinine of men has explained by a greater skeletal, muscle and bone mass compared to women [66]. But the opposite occurred in *Sprague-Dawley* and *Wistar* rats. In rodents, triglyceride levels were different. Similar finding has been obtained in a previous study [15], which is typical strain-specific difference caused by the distinct genetic background.

We didn’t fully understand the physiological differences in hematologic and biochemical analytes between male and female *Sprague-Dawley* rats as well as between rodents and humans. Factors such as genetic background, diet, temperature, hormonal cycles, metabolic rate, enzyme activities and circadian rhythms all could influence the metabolism of an organism in a dynamic manner reciprocally [67].

The finding in this study is that sex partitioning is required for many parameter reference intervals. If normal deviate z value was more than the critical z value of 5 or any of the four proportions (two at the lower and two at the upper end of the distributions) outside the common reference limits was ≥ 4.1% or ≤ 0.9%, the subgroup partitioning was presented. Other types of outcomes we considered as the combined reference intervals. We used the proportion criteria for non-normal distribution as it was more accurate than the Harris-Boyd method for such distribution [28].

Differences with other study results may be attributed to differences in measurement methods, instrument and reagent, which may affect the accuracy of the test results. Methods for all analysis were traceable to the IFCC standards and described in this study. The Sysmex XT-2000i and Olympus AU 400 have been our laboratory’s hematology and biochemistry systems for several years and have been well standardized and have documented traceability. Although the Sysmex XT-2000iV system has recently been released for use in the pharmaceutical and veterinary fields, it is useful to measure animal blood through Sysmex XT-2000i because the study has found that reproducibility and dilution linearity of Sysmex XT-2000i are high as same as human in animal blood [68]. In addition, Olympus AU 400 analyzer is also often used for clinical chemistry testing in rodents. We strictly carried out the quality assurance protocols to avoid false results due to haemolysis, contamination or operating. Exclusion criteria were used to identify healthy individuals to ensure robust calculation of reference intervals in our study. The ratio D/R proposed by Dixon was adopted to detect outliers. We decided not to exclude any data because no clear analytical or biological reasons could be demonstrated. We obtained the hematologic and biochemical values from a sufficient number of healthy *Sprague-Dawley* rats, with 500 values in total, and 250 values for each sex. The large sample size has also ensured a high degree of statistical power in determining sex-specific partitions.

The new idea of establishing common reference intervals for several laboratories that serve a homogeneous population throughout a geographic area was gaining acceptance, and multi-centric reference intervals studies were the most important development in the area of reference intervals [2, 69]. Therefore, it is necessary to establish a reference interval in each laboratory, and take part in the production of common reference intervals.

Our study has limitations. For example, we lack research of any influence of seasonal and age [70] changes on biochemical and haematological analytes in *Sprague-Dawley* rats. Besides, the number of parameters is limited. We are planning to resolve these problems in further study.

In conclusion, this report shows significant sex differences between male and female *Sprague-Dawley* rats in many hematologic and biochemical analytes. Therefore, it is necessary to partition the reference intervals based on sex. We establish the sex-specific reference intervals to more precisely evaluate the animal quality and experimental results of *Sprague-Dawley* rats in toxicology safety study.

## Supporting information

S1 Table List of hematology and biochemistry analytes with their measurement principles and analytical imprecision. (DOC)

S2 Table Application of partitioning criteria for hematologic analytes. (DOCX)

S3 Table Application of partitioning criteria for biochemical analytes. (DOC)
