# Supplementary material for: Sex-specific reference intervals of hematologic and biochemical analytes in Sprague-Dawley rats using the nonparametric rank percentile method
Source: PLoS One. 2017 Dec 20;12(12):e0189837. doi: 10.1371/journal.pone.0189837 (PMC5738108; doi:10.1371/journal.pone.0189837)
Supplement: S1 Table — (DOC) [file pone.0189837.s001.doc]

**S1 Table. List of hematology and biochemistry analytes with their measurement principles and analytical imprecision.**

| **Analyte** | **Method** | **Within-day CV** | **Between-day CV** | | | **Allowable difference*** |
| --- | --- | --- | --- | --- | --- | --- |
|  |  |  | **Level 1** | **Level 2** | |  |
| Hematocrit | Calculation using RBC and MCV | 1.06 | 1.53 | | 1.11 | 4.0 |
| Hemoglobin | Sodium lauryl sulfate hemoglobin detection method | 0.81 | 1.39 | | 0.57 | 2.0 |
| MCH | Calculation using hemoglobin and RBC | 1.36 | 0.79 | | 0.73 | 2.5 |
| MCHC | Calculation using hemoglobin and hematocrit | 1.30 | 0.69 | | 0.78 | 3.0 |
| MCV | Calculation of average volume of individual erythrocytes | 0.16 | 0.24 | | 0.33 | 2.5 |
| RBC | Impedance technology with hydro dynamic focusing method | 0.78 | 1.14 | | 0.82 | 2.5 |
| RDW-CV% | Impedance technology with hydro dynamic focusing method | 0.62 | 0.31 | | 0.89 | 15.03 |
| Platelet | Impedance technology with hydro dynamic focusing method | 1.29 | 3.97 | | 2.07 | 8.0 |
| MPV | Calculation of average volume of individual platelets | 0.91 | 0.72 | | 1.25 | 14.89 |
| WBC | Fluorescence flow cytometry method | 2.41 | 1.53 | | 1.74 | 6.0 |
| Neutrophils | Fluorescence flow cytometry method | 6.19 | 2.23 | | 3.24 | 30.03 |
| Lymphocytes | Fluorescence flow cytometry method | 3.05 | 5.07 | | 4.97 | 30.14 |
| Monocytes | Fluorescence flow cytometry method | 11.08 | 18.96 | | 13.71 | 89.53 |
| Eosinophils | Fluorescence flow cytometry method | 10.83 | 7.21 | | 6.94 | 50.70 |
| Basophils | Cell size and side scatter properties | 34.22 | 2.27 | | 2.19 | 77.95 |
| ALT | Lactate dehydrogenase method | 3.43 | 4.17 | | 4.32 | 6.0 |
| Albumin | Bromcresol green method | 1.39 | 1.62 | | 0.90 | 2.5 |
| AST | Malate dehydrogenase method | 1.44 | 1.20 | | 1.57 | 6.0 |
| Creatinine | Kinetic alkaline picrate method | 0.70 | 1.12 | | 1.23 | 4.0 |
| Glucose | Hexokinase method | 0.90 | 1.35 | | 3.30 | 7.89 |
| Total cholesterol | Cholesterol oxidase method | 0.71 | 0.85 | | 1.16 | 3.0 |
| Total protein | Biuret method | 1.12 | 1.36 | | 0.72 | 2.0 |
| Triglycerides | Glycerophosphate oxidase method | 0.94 | 1.28 | | 2.00 | 5.0 |
| Urea | Urease glutamate dehydrogenase method | 1.44 | 1.76 | | 1.18 | 3.0 |

CV, coefficient of variation.

*Allowable limits on controls are taken from the health industry standards WS/T 403-2012 and WS/T 406-2012 of the People’s Republic of China and the manufactures’s information sheet.
