# Supplementary material for: Sex-specific reference intervals of hematologic and biochemical analytes in Sprague-Dawley rats using the nonparametric rank percentile method
Source: PLoS One. 2017 Dec 20;12(12):e0189837. doi: 10.1371/journal.pone.0189837 (PMC5738108; doi:10.1371/journal.pone.0189837)
Supplement: S2 Table — (DOCX) [file pone.0189837.s002.docx]

**S2 Table. Application of partitioning criteria for hematologic analytes.**

| **Analyte** |  | **2.5th centile** | **97.5th centile** | **Kolmogorov-**  **Smirnov p-value** | **Z value /*p*%** | **Conclusion for one end** |
| --- | --- | --- | --- | --- | --- | --- |
| Hemoglobin, g/L | Male | 135 | 159 | 0.002 | 1.20%,3.59% | Partitioning |
|  | Female | 129 | 154 | 0.057* | 3.60%,0.80% |  |
|  | Combinated | 132 | 157 | 0.004 |  |  |
| Hematocrit, % | Male | 42 | 49 | 0.000 | 0.40%,4.38% | Partitioning |
|  | Female | 40 | 46 | 0.000 | 2.50%,0.40% |  |
|  | Combinated | 40 | 48 | 0.000 |  |  |
| RBC, 10^12^/L | Male | 6.71 | 8.03 | 0.071* | 6.33 | Nonpartitioning |
|  | Female | 6.33 | 7.85 | 0.200* |  |  |
|  | Combinated | 6.39 | 8.01 | 0.200* |  |  |
| RDW-CV, % | Male | 13.03 | 16.57 | 0.024 | 0.80%,4.30% | Partitioning |
|  | Female | 12.23 | 14.57 | 0.013 | 2.80%,0.80% |  |
|  | Combinated | 12.30 | 16.20 | 0.000 |  |  |
| RDW-SD, fL | Male | 29.50 | 34.55 | 0.008 | 0.40%,5.18% | Partitioning |
|  | Female | 25.90 | 31.37 | 0.031 | 4.78%,0.40% |  |
|  | Combinated | 26.25 | 34.00 | 0.000 |  |  |
| MCV, fL | Male | 58.01 | 67.00 | 0.041 | 0.40%,4.380% | Partitioning |
|  | Female | 55.21 | 64.80 | 0.200* | 4.78%,1.20% |  |
|  | Combinated | 56.00 | 66.75 | 0.090* |  |  |
| MCH, pg | Male | 18.90 | 21.20 | 0.011 | 1.20%,2.50% | Nonpartitioning |
|  | Female | 18.70 | 21.20 | 0.015 | 2.50%,2.50% |  |
|  | Combinated | 18.70 | 21.20 | 0.000 |  |  |
| MCHC,g/L | Male | 310 | 336 | 0.200* | 2.50%,0.40% | Partitioning |
|  | Female | 318 | 347 | 0.002 | 0.40%,3.98% |  |
|  | Combinated | 310 | 345 | 0.004 |  |  |
| Plateletcrit, % | Male | 0.63 | 1.14 | 0.044 | 3.59%,1.59% | Nonpartitioning |
|  | Female | 0.67 | 1.22 | 0.200* | 1.59%,3.98% |  |
|  | Combinated | 0.65 | 1.16 | 0.017 |  |  |
| PDW, fL | Male | 6.70 | 8.70 | 0.000 | 3.19%,1.99% | Nonpartitioning |
|  | Female | 6.80 | 8.90 | 0.000 | 2.39%,3.59% |  |
|  | Combinated | 6.75 | 8.85 | 0.000 |  |  |
| Platelet, 10^9^/L | Male | 878 | 1504 | 0.200* | 3.98%,1.20% | Nonpartitioning |
|  | Female | 965 | 1645 | 0.033 | 1.20%,3.98% |  |
|  | Combinated | 923 | 1580 | 0.030 |  |  |
| P-LCR,% | Male | 4.03 | 10.57 | 0.000 | 2.79%,1.99% | Nonpartitioning |
|  | Female | 4.03 | 11.60 | 0.000 | 2.79%,3.19% |  |
|  | Combinated | 4.05 | 11.20 | 0.000 |  |  |
| MPV,fL | Male | 6.63 | 8.00 | 0.000 | 2.79%,1.59% | Nonpartitioning |
|  | Female | 6.70 | 8.20 | 0.000 | 3.19%,3.59% |  |
|  | Combinated | 6.70 | 8.10 | 0.000 |  |  |
| WBC,10^9^/L | Male | 3.00 | 9.22 | 0.040 | 1.20%,4.80% | Partitioning |
|  | Female | 2.58 | 7.34 | 0.000 | 3.98%,0.40% |  |
|  | Combinated | 2.68 | 8.58 | 0.000 |  |  |
| Neutrophils, 10^9^/L | Male | 0.28 | 1.43 | 0.000 | 0.80%,4.78% | Partitioning |
|  | Female | 0.19 | 0.91 | 0.000 | 3.98%,0.80% |  |
|  | Combinated | 0.20 | 1.18 | 0.000 |  |  |
| Lymphocytes, 10^9^/L | Male | 2.45 | 7.66 | 0.010 | 2.00%,5.18% | Partitioning |
|  | Female | 2.09 | 6.39 | 0.000 | 3.59%,0.40% |  |
|  | Combinated | 2.17 | 7.05 | 0.000 |  |  |
| Monocytes, 10^9^/L | Male | 0.17 | 0.76 | 0.000 | 0.40%,4.78% | Partitioning |
|  | Female | 0.08 | 0.43 | 0.004 | 4.80%,0.40% |  |
|  | Combinated | 0.11 | 0.67 | 0.000 |  |  |
| Eosinophils, 10^9^/L | Male | 0.03 | 0.18 | 0.000 | 2.50%,1.99% | Nonpartitioning |
|  | Female | 0.03 | 0.22 | 0.000 | 2.50%,3.12% |  |
|  | Combinated | 0.03 | 0.21 | 0.000 |  |  |
| Basophils, 10^9^/L | Male | 0 | 0.03 | 0.000 | 2.50%,2.78% | Nonpartitioning |
|  | Female | 0 | 0.01 | 0.000 | 2.5%,1.59% |  |
|  | Combinated | 0 | 0.02 | 0.000 |  |  |
| Neutrophils, % | Male | 6.14 | 22.95 | 0.000 | 0.80%,3.59% | Partitioning |
|  | Female | 4.27 | 18.48 | 0.000 | 3.98%,1.59% |  |
|  | Combinated | 4.54 | 20.84 | 0.000 |  |  |
| Lymphocytes, % | Male | 69.68 | 86.89 | 0.000 | 3.59%,0.40% | Partitioning |
|  | Female | 71.77 | 89.94 | 0.001 | 1.59%,4.78% |  |
|  | Combinated | 70.66 | 89.34 | 0.002 |  |  |
| Monocytes, % | Male | 3.77 | 10.82 | 0.003 | 0.80%,3.59% | Partitioning |
|  | Female | 2.10 | 9.34 | 0.000 | 4.78%,1.20% |  |
|  | Combinated | 2.84 | 10.54 | 0.000 |  |  |
| Eosinophils, % | Male | 0.54 | 3.39 | 0.000 | 4.38%,1.59% | Partitioning |
|  | Female | 0.84 | 4.29 | 0.000 | 1.20%,2.79% |  |
|  | Combinated | 0.64 | 4.14 | 0.000 |  |  |
| Basophils, % | Male | 0.04 | 0.51 | 0.000 | 2.50%,2.79% | Nonpartitioning |
|  | Female | 0.04 | 0.42 | 0.000 | 2.50%,2.50% |  |
|  | Combinated | 0.04 | 0.44 | 0.000 |  |  |

*Kolmogorov-Smirnov p-value＞0.05 indicates that the data are not skewed and are a normal distribution. Conversely, if Kolmogorov-Smirnov p-value＜0.05 indicates that the data are found to be skewed and are non-normal distributions.

If z value ≥7.22 or any of the four proportions (two at the lower and two at the upper end of the distributions) outside the common reference limits is ≥4.1% or ≤0.9%, the subgroup partitioning is recommended.
