# Supplementary material for: Sex-specific reference intervals of hematologic and biochemical analytes in Sprague-Dawley rats using the nonparametric rank percentile method
Source: PLoS One. 2017 Dec 20;12(12):e0189837. doi: 10.1371/journal.pone.0189837 (PMC5738108; doi:10.1371/journal.pone.0189837)
Supplement: S3 Table — (DOC) [file pone.0189837.s003.doc]

**S3 Table. Application of partitioning criteria for biochemical analytes.**

|  | | | | | | |
| --- | --- | --- | --- | --- | --- | --- |
| **Analyte** |  | **2.5th centile** | **97.5th centile** | **Kolmogorov-Smirnov p-value** | **Z value /*p*%** | **Conclusion for one end** |
| ALT, U/L | Male | 19 | 47 | 0.000 | 0.80%,5.18% | Partitioning |
|  | Female | 14 | 30 | 0.000 | 4.78%,0.40% |
|  | Combinated | 16 | 42 | 0.000 |  |
| Albumin, g/L | Male | 27.23 | 34.52 | 0.092* | 2.62 | Nonpartitioning |
|  | Female | 26.63 | 34.65 | 0.200* |
|  | Combinated | 26.85 | 34.55 | 0.020 |  |
| AST, U/L | Male | 61 | 139 | 0.002 | 1.99%,2.50% | Nonpartitioning |
|  | Female | 58 | 134 | 0.000 | 3.59%,1.99% |
|  | Combinated | 60 | 139 | 0.000 |  |
| Creatinine, μmol/L | Male | 32.36 | 47.90 | 0.000 | 4.38%,0.80% | Partitioning |
|  | Female | 34.91 | 59.67 | 0.000 | 1.20%,4.78% |
|  | Combinated | 33.21 | 57.70 | 0.000 |  |
| Glucose, mmol/L | Male | 4.46 | 7.24 | 0.200* | 14.29 | Partitioning |
|  | Female | 5.31 | 8.01 | 0.200* |
|  | Combinated | 4.77 | 7.79 | 0.200* |  |
| Total protein, g/L | Male | 51.96 | 64.75 | 0.200* | 1.32 | Nonpartitioning |
|  | Female | 50.66 | 64.47 | 0.200* |
|  | Combinated | 51.10 | 64.55 | 0.200* |  |
| Total cholesterol, mmol/L | Male | 0.68 | 1.77 | 0.050 | 7.93 | Partitioning |
|  | Female | 0.81 | 2.03 | 0.200* |
|  | Combinated | 0.75 | 1.96 | 0.032 |  |
| Triglycerides, mmol/L | Male | 0.23 | 0.99 | 0.000 | 0.40%,5.18% | Partitioning |
|  | Female | 0.16 | 0.89 | 0.000 | 4.38%,0.80% |
|  | Combinated | 0.18 | 0.90 | 0.000 |  |
| Urea, mmol/L | Male | 4.32 | 8.97 | 0.024 | 3.59%,0.80% | Partitioning |
|  | Female | 5.56 | 12.67 | 0.000 | 0.40%,4.78% |
|  | Combinated | 4.54 | 11.79 | 0.000 |  |

*Kolmogorov-Smirnov p-value＞0.05 indicates that the data are not skewed and are a normal distribution. Conversely, if Kolmogorov-Smirnov p-value＜0.05 indicates that the data are found to be skewed and are non-normal distributions.

If z value ≥7.22 or any of the four proportions (two at the lower and two at the upper end of the distributions) outside the common reference limits is ≥4.1% or ≤0.9%, the subgroup partitioning is recommended.
